# Antibiotics in critically ill patients: a systematic review of the pharmacokinetics of β-lactams

**DOI:** 10.1186/cc10441

**Published:** 2011-09-13

**Authors:** Joao Gonçalves-Pereira, Pedro Póvoa

**Affiliations:** 1 Polyvalent Intensive Care Unit, São Francisco Xavier Hospital, Estrada do Forte do Alto do Duque, 1449-005 Lisboa, Portugal; 2 CEDOC, Faculty of Medical Sciences, New University of Lisbon, Campo dos Mártires da Pátria, 130, 1169-056 Lisboa, Portugal

**Keywords:** administration, dosage, β-lactam antibiotics, microdialysis, pharmacodynamics, pharmacokinetics, ICU

## Abstract

**Introduction:**

Several reports have shown marked heterogeneity of antibiotic pharmacokinetics (PK) in patients admitted to ICUs, which might potentially affect outcomes. Therefore, the pharmacodynamic (PD) parameter of the efficacy of β-lactam antibiotics, that is, the time that its concentration is above the bacteria minimal inhibitory concentration (T > MIC), cannot be safely extrapolated from data derived from the PK of healthy volunteers.

**Methods:**

We performed a full review of published studies addressing the PK of intravenous β-lactam antibiotics given to infected ICU patients. Study selection comprised a comprehensive bibliographic search of the PubMed database and bibliographic references in relevant reviews from January 1966 to December 2010. We selected only English-language articles reporting studies addressing β-lactam antibiotics that had been described in at least five previously published studies. Studies of the PK of patients undergoing renal replacement therapy were excluded.

**Results:**

A total of 57 studies addressing six different β-lactam antibiotics (meropenem, imipenem, piperacillin, cefpirome, cefepime and ceftazidime) were selected. Significant PK heterogeneity was noted, with a broad, more than twofold variation both of volume of distribution and of drug clearance (Cl). The correlation of antibiotic Cl with creatinine clearance was usually reported. Consequently, in ICU patients, β-lactam antibiotic half-life and T > MIC were virtually unpredictable, especially in those patients with normal renal function. A better PD profile was usually obtained by prolonged or even continuous infusion. Tissue penetration was also found to be compromised in critically ill patients with septic shock.

**Conclusions:**

The PK of β-lactam antibiotics are heterogeneous and largely unpredictable in ICU patients. Consequently, the dosing of antibiotics should be supported by PK concepts, including data derived from studies of the PK of ICU patients and therapeutic drug monitoring.

## Introduction

Infection and sepsis, whether community- or hospital-acquired, are important causes of morbidity and mortality in ICU patients [1,2]. Despite all of the research, sepsis therapy continues to depend on supportive management of the different organ dysfunctions and failures and on specific therapy for infection with timely and appropriate antibiotics and/or focus control.

The β-lactam antibiotics, because of their large antimicrobial spectrum and low toxicity, are among the first-line therapies for critically ill patients, especially when a Gram-negative infection is suspected. However, the efficacy of antibiotics is not easily evaluated, since the clinical response is usually unnoticeable before 48 hours of therapy [3]. Moreover, the unavailability of routine therapeutic drug monitoring for the great majority of these drugs makes it difficult to distinguish clinical failure due to underdosing from lack of *in vivo *organism susceptibility.

Considerable evidence demonstrates that free drug time above bacteria minimal inhibitory concentration (*f *T > MIC) is the measure of drug exposure most closely linked to the ability of β-lactam antibiotics to kill the target bacteria [4]. T > MIC is dependent on the half-life (T_1/2_) of β-lactam antibiotics and their serum concentration.

The serum concentration of an antibiotic depends on the dose delivered, its bioavailability and its volume of distribution (*V*_d_). *V*_d _is a mathematical construct and refers to the size of a compartment necessary to account for the total amount of the drug, assuming that its concentration in the whole body is equal to that measured in plasma. Drugs that distribute essentially in the extracellular fluid (mainly hydrophilic) have low *V*_d_, whilst drugs that have rapid cellular uptake (lipophilic) have high *V*_d_[5,6].

**Figure 1:**
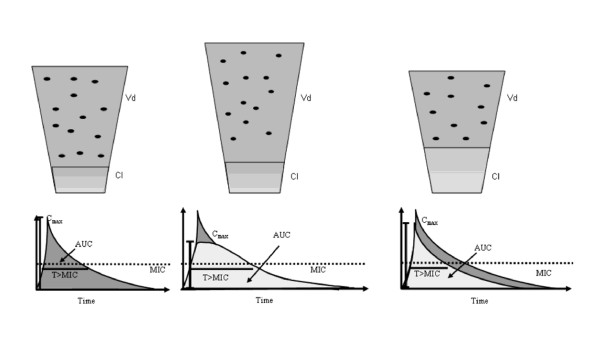
**ICU patients present pharmacokinetic changes of antibiotics that may alter bacterial exposure**. Concentration-time curve of antibiotics in healthy volunteers (left panel). A large volume of distribution (*V*_d_) (middle panel) is often present in ICU patients, leading to decreased maximum concentration (C_max_) but a longer half-life (T_1/2_) and eventually higher time that the antibiotic concentration is above the bacteria minimum inhibitory concentration (T > MIC). The antibiotic area under the concentration time curve (AUC) remains virtually the same. An increase in drug clearance (Cl) (right) is associated with decreases in AUC, T_1/2 _and T > MIC. Straight dotted lines-bacteria minimum inhibitory concentration.

Both *V*_d _and drug clearance (Cl) may be increased in ICU patients [7]. Therapeutic procedures, notably large-volume and blood products infusions, positive pressure ventilation, surgical procedures, capillary leak and reduction in albumin serum concentration all contribute to alter the concentration-time relationship of many drugs. A rise in the *V*_d_, although it reduces drug concentration, might proportionally increase T_1/2_, since T_1/2 _= *V*_d_/(Cl × 0.693) [7]. On the contrary, a high Cl may reduce the exposure of antibiotics to bacteria (Figure 1).

Renal Cl may be increased in septic patients because of increased renal blood flow. This has recently been shown to be a common finding in ICU patients, particularly surgical and trauma patients [8] but also septic medical patients [9]. Besides, in the study by Baptista *et al*. [9], the authors showed that commonly used formulas used to calculate glomerular filtration rate usually underestimate creatinine (Cr) Cl. Consequently, these authors recommended direct Cr Cl measurement.

Moreover in ICU patients, maldistribution of blood flow in the microcirculation [10], namely, in patients in septic shock, may further decrease the drug concentration in the infected tissue [11]. These pharmacokinetic (PK) changes are sometimes influenced by the clinical course of the infection itself [12]. Consequently, PK parameters measured in healthy volunteers may not correctly predict concentrations in septic ICU patients, particularly early in the course of a severe infection [13,14].

Ideally, individualized dosing strategies should account for the altered PK and pathogen susceptibility in each patient. Despite the fact that some studies addressed this issue, this information had not yet been aggregated. Furthermore, β-lactam antibiotic PK are rarely analysed outside clinical trials. Therefore, we performed a systematic review of studies that addressed the PK parameters of β-lactam antibiotics in ICU patients to assess the relationship between dose and schedule of β-lactam antibiotics and their adequacy according to pharmacodynamic (PD) end points. We also reviewed studies assessing the concentrations of β-lactam antibiotics in different tissues. Our primary intention was to aggregate PK information in this particular population and to contribute to the design of individualized dosing regimens of these drugs.

We also included studies that involved the development of PD models using PK of ICU patients and bacterial MICs. These techniques allow the calculation of the presumed T > MIC and therefore the percentage of patients in which the antibiotic will achieve its PD target: that is, the antibiotic's probability of target attainment (PTA) [15,16]. The cumulative fraction of response (CFR) is calculated by multiplying the PTA obtained for each MIC by the MIC distribution according to a microbiological database [16].

## Materials and methods

**Figure 2:**
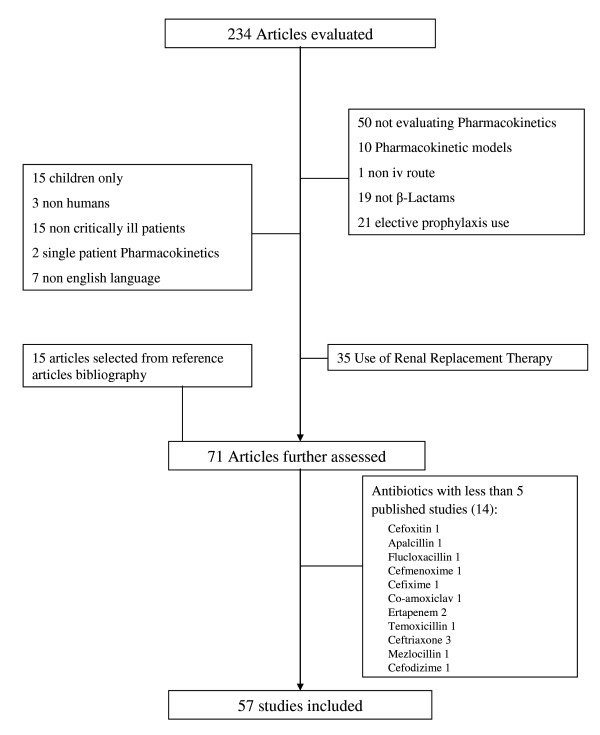
**Flow diagram illustrating the selection of studies included in this review**.

The data for this review were identified by a search of PubMed (January 1966 to December 2010) as well as bibliographic references from relevant articles, including reviews on this subject and all selected studies. The search terms used were 'antibiotic' or 'carbapenem' or 'penicillins' or 'cephalosporins', and 'intensive care' or 'critically ill' or 'critical care' or 'severe sepsis' or 'septic shock', and 'pharmacokinetics' or 'pharmacodynamics'. All relevant studies in the English-language literature that described antibiotic PK in critically ill patients were assessed (Figure 2).

Only studies that described PK of antibiotics given intravenously to infected patients were selected. Studies referring to prophylactic antibiotics or to PK in patients under any type of renal replacement therapy were excluded. In fact, these studies are mainly directed to the measurement of Cl during renal replacement therapy to determine the ideal antibiotic dose and therefore are not easily compared with studies addressing the intrinsic PK of ICU patients. Furthermore, a full revision of those studies has recently been published [17].

**Figure 3:**
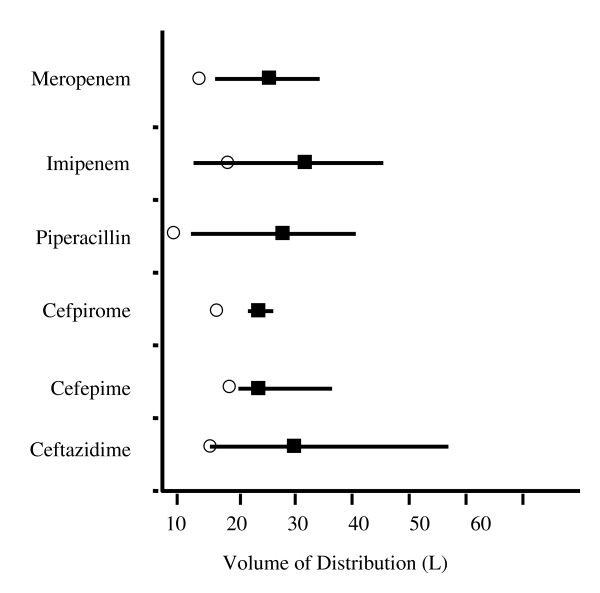
**Heterogeneity of volume of distribution in litres of β-lactam antibiotics in ICU patients**. Open circles: volume of distribution in healthy volunteers [44,51,89-92]; filled squares: weighted means of volume of distribution in the studies; straight lines: ranges of the means of volume of distribution in the studies.

For the purpose of our systematic review, we analysed only studies of antibiotics with at least five published references. This threshold of five referenced studies was arbitrarily chosen so that we could derive more representative and consistent data on the PK of each antibiotic. The weighted mean of the *V*_d _was calculated so that we could present a graphic representation of each analysed antibiotic (Figure 3).

## Results

A total of 57 studies assessing an aggregate of six different β-lactam antibiotics were selected.

### Carbapenem

#### Meropenem

Several studies have addressed meropenem PK in ICU septic patients. High *V*_d _and Cl have usually been reported, as well as a low binding fraction: < 10% [18]. Consequently, a large heterogeneity of PK parameters was found, exceeding a twofold variation (Table 1 and Figure 3). The larger reported *V*_d_, a mean of 34.4 L, was noted on the second day of therapy in eight ventilator-associated pneumonia (VAP) patients [19] with a mean body weight of 73 kg. In a Thai VAP population (*N *= 9) with a lower mean body weight (only 54.2 kg), the mean *V*_d _was 6.0 L despite also being measured after 48 hours of therapy with meropenem [20]. This supports the hypothesis of a potential relationship between body weight and *V*_d_.

**Table 1:** Pharmacokinetic parameters of β-lactam antibiotics^a^

	PK parameters				Study	
Antibiotic drug classes and drugs	*V*_d_, L	Cl, L/hour	T_1/2_, hours	Patient demographics	**Study types **[93]	References
Carbapenems						
Meropenem	21.2 ± 4.7^b^	11.3 ± 4^b^	1.4 ± 0.4^b^	*N *= 11 Age 63.1 years [23 to 81] Mild to severe intraabdominal sepsis	Descriptive	Lovering *et al*., 1995 [22]
Meropenem	26.6 ± 3.2^c^	9.4 ± 1.2^c^	2.0	*N *= 15 Age 55.3 ± 14.3 years Severe sepsis	Randomized, controlled cross-over	Thalhammer *et al*., 1999 [27]
Meropenem	34.4 ± 15.9	11 ± 4.3	0.4 ± 0.12	*N *= 8 Age 55 ± 8 years VAP	Descriptive	de Stoppelaar *et al*., 2000 [19]
Meropenem	19.7 ± 5	7.3 ± 3.1	3.1 ± 1.5	*N *= 14 Age 73.3 ± 8.1 years Severe sepsis	Descriptive	Kitzes-Cohen *et al*., 2002 [21]
Meropenem	16.0 ± 3.7^d^	8.5 ± 3.2^d^	1.4 ± 0.6^d^	*N *= 9 Age 39.6 ± 15.7 years VAP	Not randomized, controlled cross-over	Jaruratanasirikul *et al*., 2005 [20]
Imipenem	Imipenem 17.7 ± 4	Imipenem 7.0 ± 2.5	Imipenem 2 ± 0.3	Imipenem *N *= 10 Age 65 ± 19 years	Randomized, parallel controlled	Novelli *et al*., 2005 [29]
Meropenem	Meropenem 27.1 ± 7.7	Meropenem 11.5 ± 3.1	Meropenem 2.1 ± 0.5	Meropenem *N *= 10 Age 67 ± 19 years Severe sepsis		
Meropenem	23.8 ± 4.9	6.7 ± 4.2	3.7 ± 1.9	*N *= 6 Age 65.7 ± 11.2 years Peritonitis	Descriptive	Karjagin *et al*., 2008 [25]
Meropenem	22.7	13.6 ± 1.3	NR	*N *= 10 Age range 48 to 63 years Severe sepsis	Randomized, parallel controlled	Roberts *et al*., 2009 [24]
Meropenem	Meropenem 30.1 [21.7 to 53.9]^e^	Meropenem 8 [5 to 10.99^e^	Meropenem 2.1 [1.7 to 3.4]	Meropenem *N *= 16	Cross-sectional	Taccone *et al*., 2010 [23]
Piperacillin	Piperacillin 26.6 [20.3 to 30.1]^e^	Piperacillin 8.4 [5.5 to 18.1]^e^	Piperacillin 2.6 [1.5 to 3.8]	Piperacillin *N *= 27		
Ceftazidime	Ceftazidime 33.6 [25.2 to 49.7]^e^	Ceftazidime 3.8 [2.5 to 5.5]^e^	Ceftazidime 5.8 [4.1 to 7.4]	Ceftazidime *N *= 18		
Cefepime	Cefepime 25.2 [23.1 to 30.8]^e^	Cefepime 5.5 [4.6 to 8.4]^e^	Cefepime 3.4 [2.3 to 5.3]	Cefepime *N *= 19 All patients: median age 63 years Severe sepsis or septic shock		
Imipenem	31.4 ± 11.7	14.4 ± 4.5	1.6 ± 1.3	*N *= 10 Age 44 ± 12.2 years Severe sepsis	Descriptive	McKindley *et al*., 1996 [34]
Imipenem	18.5	6.3 ± 0.8	2.0	*N *= 6 Age 63.5 ± 16.7 years Severe sepsis	Not randomized, parallel, controlled	Tegeder *et al*., 2002 [32]
Imipenem	45.5 ± 47.2	12.1 ± 12.0	2.9 ± 1.7	*N *= 50 Age 45.2 ± 17 years Presumed Gram-negative sepsis	Cross-sectional	Belzberg *et al*., 2004 [28]
Imipenem	12.2 ± 9.9^f^	12.3 ± 4.2	NR	*N *= 20 Age 60.5 years VAP	Randomized, parallel, controlled	Sakka *et al*., 2007 [31]
Imipenem	27.2 ± 6.5	13.3 ± 5.2	1.4 ± 0.2	*N *= 6 Age 53.3 ± 19.9 years Severe sepsis	Not randomized, parallel, controlled	Dahyot *et al*., 2008 [33]
Imipenem	16.7 ± 5.3^g^	8.7 ± 5.3^g^	1.5 ± 0.7^g^	*N *= 9 Age 63.3 ± 14.9 years VAP	Not randomized, controlled, cross-over	Jaruratanasirikul and Sudsai, 2009 [30]
Penicillins						
Piperacillin	25.0 ± 17.2	23.8 ± 17.2	1.5 ± 2.1	*N *= 11 Age 43.6 ± 15.9 years Surgical patients	Descriptive	Shikuma *et al*., 1990 [36]
Piperacillin	19.5 ± 3.4^b^	8.4 ± 1.4^b^	1.8 ± 0.3^b^	*N *= 10 Age 37.7 ± 2.8 years Burn patients	Descriptive	Bourget *et al*., 1996 [38]
Piperacillin	40.7 ± 8.7	8.2 ± 2	4.1 ± 1.3	*N *= 6 Age 64 ± 7 years Septic shock	Not randomized, parallel, controlled	Joukhadar *et al*., 2001 [44]
Piperacillin	34.6 ± 6.8^c^	11.8 ± 4.3^c^	2.4 ± 1.2^c^	*N *= 7 Age range 45 to 76 years Severe sepsis	Not randomized, controlled, cross-over	Langgartner *et al*., 2007 [39]
Piperacillin	11.7^f^	17.2	0.4	*N *= 13 Age 37.5 ± 19.4 years Severe sepsis	Randomized, parallel, controlled	Roberts *et al*., 2009 [45]
Cephalosporins						
Cefpirome	23.6 ± 8.0	8.0 ± 3.0	2.2 ± 0.5	*N *= 9 Age 31 years [19 to 53] Severe sepsis	Not randomized, parallel, controlled	Jacolot *et al*., 1999 [47]
Cefpirome	26.4 ± 7.9	8.8 ± 3.4	3.1 ± 1.2	*N *= 12 Age 41.2 ± 19 years Severe sepsis	Descriptive	Lipman *et al*., 2001 [48]
Cefpirome	25.9 ± 7.1	4.5 ± 0.7	3.3 ± 0.5	*N *= 12 Age 67.2 ± 8.1 years Severe sepsis or septic shock	Not randomized, parallel, controlled	Joukhadar *et al*., 2002 [52]
Cefpirome	21.9 ± 4.5	4.8 ± 1.6	3.1 ± 0.9	*N *= 11 Age 66 ± 8 years Severe sepsis	Not randomized, parallel, controlled	Sauermann *et al*., 2005 [51]
Cefepime	32.6 ± 17.5	7.5 ± 3.1	3.5 ± 1.1	*N *= 7 Age 73.7 ± 4.9 years Severe sepsis	Descriptive	Kieft *et al*., 1993 [53]
Cefepime	21.8 ± 5.1	7.6 ± 2.0	3 ± 1.2	N = 13 Age 55 years Severe sepsis	Descriptive	Lipman *et al*., 1999 [56]
Cefepime	36.1 ± 11.8	8.8 ± 2.4	2.8 ± 0.6	*N *= 12 Age 41 ± 13 years Burn patients	Descriptive	Bonapace *et al*., 1999 [57]
Cefepime	26.0^b^	9.1 ± 1.5^b^	2.5 ± 0.6^b^	*N *= 6 Age 39.8 ± 11.3 years Burn patients	Descriptive	Sampol *et al*., 2000 [61]
Cefepime	Cefepime 19.6	Cefepime 7.1 ± 3.6	Cefepime 2.9 ± 3.2	Cefepime *N *= 13 Age 48.2 ± 21.2 years	Cross-sectional	Conil *et al*., 2007 [54]
Ceftazidime	Ceftazidime 28.8	Ceftazidime 7.5 ± 3.8	Ceftazidime 3.1 ± 2.1	Ceftazidime *N *= 17 Age 62.9 ± 22.4 years Burn patients		
Cefepime	28.7 ± 13.3^d^	9.1 ± 5.6^d^	4.3 ± 4.2	*N *= 21 Age 55.1 years (median) Nosocomial pneumonia	Cross-sectional	Chapuis *et al*., 2010 [55]
Ceftazidime	24.5	7.5	2.1	*N *= 16 Age range 18 to 70 years *Pseudomonas *infection	Descriptive	Rondanelli *et al*., 1986 [64]
Ceftazidime	49.3 ± 18.2^e^	15.5 ± 2.5^e^	1.8 ± 0.5^e^	*N *= 5 Age 52.3 years [21 to 69] VAP	Not randomized, controlled, cross-over	Langer *et al*., 1991 [76]
Ceftazidime	29.5 ± 8.7	4.2 ± 1.9	6.1 ± 2.5	*N *= 12 Age 60 ± 13 years VAP	Not randomized, controlled, cross-over	Bressolle *et al*., 1992 [77]
Ceftazidime	18.9 ± 9^c^	5.1^c^	3.5 ± 1.6^c^	*N *= 12 Age 57 ± 12 years Suspected Gram-negative sepsis	Not randomized, controlled, cross-over	Benko *et al*., 1996 [67]
Ceftazidime	15.0 ± 4.3	5.2 ± 2.2	1.3 ± 1.2	*N *= 10 Age 48 ± 15.1 years Severe sepsis	Descriptive	Young *et al*., 1997 [65]
Ceftazidime	56.9 ± 25.9	9.1 ± 4.8	4.8 ± 1.9	*N *= 15 Age 59.3 ± 14.6 years Severe sepsis	Descriptive	Gómez *et al*., 1999 [66]
Ceftazidime	22.9 [11.8 to 28.1]	2.8 [0.2 to 7.8]	7.7 [2 to 44.7]	*N *= 21 Age range 27 to 73 years Melioidosis	Not randomized, parallel, controlled	Angus *et al*., 2000 [71]
Ceftazidime	25.6 ± 11.2^c^	11.0 ± 5.3^c^	1.7 ± 0.7^c^	*N *= 14 Age 36.1 ± 12.8 years Gram-negative nosocomial pneumonia	Not randomized, parallel, controlled	Hanes *et al*., 2000 [70]
Ceftazidime	19.6 [14 to 28]^c, e^	5.1 [2.3 to 8.9]^c^	4.2 [1.3 to 12.3]^c^	*N *= 6 Age 64 years [42 to 87] Surgical peritonitis	Not randomized, parallel, controlled	Buijk *et al*., 2002 [74]

^a^Cl: clearance; NR: not reported; PK: pharmacokinetics; T_1/2_: half-life; VAP: ventilator-associated pneumonia; *V*_d_: volume of distribution. ^b^first-day PK; ^c^PK after bolus dosing; ^d^PK after 1-g bolus dosing; ^e^for 70 kg; ^f^central compartment; ^g^PK after 500-mg bolus dosing. Except where otherwise indicated, data are means, means ± standard deviations or medians [interquartile ranges].

Meropenem Cl ranged from a mean of 4.7 L/hour to a mean of 15.4 L/hour and was generally found to be closely correlated to Cr Cl. In fact, in patients with severe sepsis, the six patients with the lower Cr Cl (< 50 mL/minute) had the higher T > MIC and area under the concentration time curve (AUC) (230.2 mg × hour/L vs. 119.4 mg × hour/L; *P *= 0.001), despite a reduction in the dose administered, from 1 g every 8 h (tid) to 1 g every 12 h (bid) [21].

One study addressed the variability of individual meropenem PK between the first and fourth days of therapy in 11 surgical patients [22]. Despite an increase in Cr Cl from a mean of 63.9 to 79.1 mL/minute during the study period, meropenem *V*_d_, Cl and AUC remain unchanged. Nevertheless, in another study, by Taccone *et al*. [23], predefined targets were reached in only 75% of severe sepsis and septic shock patients after the first dose of 1 g of meropenem (Table 2), despite the inclusion of patients with acute renal failure (22%) who did not receive renal replacement therapy. These authors concluded that PK changes induced by sepsis were largely unpredictable and that none of the evaluated clinical parameters were predictive of PK adequacy: namely, age, severity, presence of shock, use of vasopressors and mechanical ventilation. Also, Roberts *et al*. [24] showed that the *V*_d _in patients with severe sepsis had great variability, both in the same patient (especially the central compartment: roughly 45%) and in different patients (nearly 27%). In their study, despite the fact that all patients had a serum Cr < 1.36 mg/dL, the meropenem Cl variability (in the same patient and between patients) still ranged between 10% and 20%.

**Table 2:** Pharmacodynamic targets of β-lactam antibiotics^a^

Antibiotics	PD targets	Percentage of patients achieving targets	References
Meropenem, 1 g tid or 3 g/day CI	40% *f *T > MIC, with *f *assumed to be 98%. CFR according to Mystic database	PTA for MIC = 2 mg/L: bolus 100%, CI 100%	Roberts *et al*., 2009 [24]
		PTA for MIC = 8 mg/L: bolus 70%, CI 100%	
		CFR for EC: bolus 100%, CI 100%	
		CFR for PA: bolus 40.6%, CI 100%	
Ceftazidime, 2 g	70% T > 4 × EUCAST breakpoint of PA	28%	Taccone *et al*., 2010 [23]
Cefepime, 2 g	70% T > 4 × EUCAST breakpoint of PA	16%	
Meropenem, 1 g	40% T > 4 × EUCAST breakpoint of PA	75%	
Piperacillin/tazobactam, 4.5 g	50% T > 4 × EUCAST breakpoint of PA	44%	
Imipenem 1 g tid or 2 g/day CI	40% *f *T > MIC, with *f *assumed to be 80%	MIC = 2 mg/L bolus dosing 88%, CI 100%	Sakka *et al*., 2007 [31]
		MIC = 4 mg/L bolus 75%, CI 86%	
Piperacillin/tazobactam 4.5 g qid or 13.5 g CI	50% *f *T > MIC. CFR according to Mystic database	PTA for MIC = 0.25 mg/L bolus 79.2%, CI 100%	Roberts *et al*., 2009 [46]
		PTA for MIC = 1 mg/L bolus 60%, CI 100%	
		CFR for 18 g/day: bolus 53.4%, CI 92.5%	
		CFR for 13.5 g/day: bolus 40%, CI 92.4%	
Cefpirome 2 g bid	60% T > MIC	PTA for MIC = 4 mg/L: bolus 60%, CI (4 g/day) 100%	Lipman *et al*., 2001 [48]
		PTA for MIC = 16 mg/L: bolus 10%, CI (4 g/day) 50%	
Cefpirome 2 g tid	60% T > MIC plasma and tissue	PTA for MIC = 4 mg/L: plasma 100%, tissue 100%	Sauermann *et al*., 2005 [51]
		PTA for MIC = 16 mg/L: plasma 87.5%, tissue 75%	
Cefpirome 2 g bid	65% *f *T > MIC, with *f *assumed to be 90%. CFR according to EUCAST database	CFR for EC: bolus 99.9%, CI (4 g/day) 100%	Roos *et al*., 2007 [50]
		CFR for PA: bolus 56.1%, CI (4 g/day) 84.4%	
Cefepime 2 g	60% T > MIC MIC = 8 mg/L (NCCLS break point of PA)	PTA with 1 g bid 45% PTA with 2 g bid 68%	Bonapace *et al*., 1999 [57]
		PTA for MIC = 4 mg/L: 1 g bid 68%, 2 g bid 89%	
Cefepime 2 g	65% *f *T > MIC, with *f *assumed to be 90%. CFR according to Queensland Health Pathology Service	CFR for EC: 2 g bid 78.9%, CI (4 g/day) 96.9%	Roos *et al*., 2006 [60]
		CFR for PA: 2 g bid 54%, CI (4 g/day) 91.7%	
Ceftazidime 1 g every 4 hours	100% T > 4 × MIC (isolated pathogens; if negative cultures 100% T > 16 mg/L)	Ceftazidime 47.8% PTA with 1 g every 3 hours 88.2%	Conil *et al*., 2007 [54]
Cefepime 2 g tid		Cefepime 20% PTA with 1 g every 4 hours 88.2%	
Cefepime 2 g tid	50% *f *T > MIC, with *f *assumed to be 85%	PTA for MIC = 8 mg/L 91.8%	Nicasio *et al*., 2009 [59]
		PTA for MIC = 32 mg/L 50.3%	
Cefepime 2 g (each 12 to 36 hours)	50% T > MIC MIC = 8 mg/L	First dose 67%; steady-state 44%	Chapuis *et al*., 2010 [55]
Ceftazidime 2 g tid	100% T > 5 × MIC MIC = 8 mg/L (PA break point)	10%	Young *et al*., 1997 [65]
		PTA for CI (6 g/day) 60%	
Ceftazidime 2 g tid or 6 g/day CI	100% T > 5 × MIC MIC = 8 mg/L (PA break point)	Bolus 20%	Lipman *et al*., 1999 [68]
		CI 100%	
Ceftazidime 1.5 g tid or 4.5 g/day CI	T > 4 × MIC plasma and peritoneum (isolated pathogens)	Plasma: bolus dosing 100%, CI 100%	Buijk *et al*., 2002 [74]
		Peritoneum: bolus 88%, CI 100%	
Ceftazidime 2 to 6 g/day CI	100% T > 5 × MIC MIC = 8 mg/L (PA break point) Target concentration 40 ± 10 mg/L	35.9%	Aubert *et al*., 2010 [72]
		**Percentage of time on target (mean)**	
Meropenem 2 g tid or 3 g CI	T > MIC (isolated susceptible pathogens)	Bolus T = 100%; CI T = 100%	Thalhammer *et al*., 1999 [27]
Meropenem 1 g tid	T > MIC (isolated pathogens)	T = 90.8%	de Stoppelaar *et al*, 2000 [19]
		T > 4 × MIC T = 52%	
Meropenem 1 g bid or 1 g tid	T > MIC (isolated pathogens)	T = 80.9% (Cr Cl > 50 mL/minute; 1 g tid)	Kitzes-Cohen *et al*, 2002 [21]
		T = 91.7% (Cr Cl < 50 mL/minute; 1 g bid)	
Imipenem 1 g tid	T > MIC (isolated sensitive [MIC ≤ 2 mg/L] pathogens)	T = 100%; T > 4 × MIC T = 87.5%	Novelli *et al*., 2005 [29]
Meropenem 1 g tid	T > MIC (isolated sensitive [MIC ≤ 2 mg/L] pathogens)	T = 100%; T > 4 × MIC T = 87.5%	
Meropenem 1 g tid (bolus or 3-hour infusion) or 2 g tid (3-hour infusion)	T > MIC	For MIC = 1 mg/L: 1 g tid bolus T = 74.7%, 1 g tid 3 hours T = 93.6%, 2 g tid 3 hours T = 98.6%s	Jaruratanasirikul *et al*., 2005 [20]
		For MIC = 16 mg/L: 1 g tid bolus T = 28.3%, 1 g tid 3 hours T = 37.8%, 2 g tid 3 hours T = 57.9%	
Meropenem 1 g tid	T > MIC	For MIC = 4 mg/L: plasma T = 87%, peritoneum T = 87%	Karjagin *et al*., 2008 [25]
		For MIC = 16 mg/L: plasma T = 55%, peritoneum T = 43%	
Imipenem 500 mg qid (30 minutes or 2-hour infusion) or 1 g qid (2-hour infusion)	T > MIC	For MIC = 1 mg/L: 500 mg qid 30 minutes T = 64.7%, 500 mg qid 2 hours T = 76.5%, 1 g qid 2 hours T = 93.4%	Jaruratanasirikul and Sudsai, 2009 [30]
		For MIC = 4 mg/L: 500 mg qid 30 minutes T = 20.3%, 500 mg qid 2 hours T = 17.7%, 1 g qid 2 hours T = 60.3%	
Piperacillin 3 g qid or 8 g/day CI	T > MIC	For MIC = 16 mg/L: bolus dosing T = 62%, CI T = 100%	Rafati *et al*., 2006 [40]
		For MIC = 32 mg/L: bolus T = 39%, CI T = 65%	
Cefepime 2 g bid	T > MIC MIC = 7 mg/L (MIC_90 _of PA)	T = 80%	Kieft *et al*., 1993 [53]
Ceftazidime 2 g tid or 3 g/day CI	T > MIC MIC = 4 mg/L (MIC of one isolated PA)	Bolus T = 92%; CI T = 100%	Benko *et al*., 1996 [67]
Ceftazidime 2 g tid or 60 mg/kg/day CI	T > MIC (isolated pathogens)	Bolus T = 92.9%; CI T = 100%	Hanes *et al*., 2000 [70]

^a^AB: *Acinetobacter baumanii*; bid: dose every 12 hours; CFR: cumulative fraction of response; CI: continuous infusion; Cr Cl: creatinine clearance; EC: *Escherichia coli*; EUCAST: European Committee on Antimicrobial Susceptibility Testing; *f*: free drug fraction; KP: *Klebsiella pneumoniae*; MIC: minimal inhibitory concentration; MIC_90_: 90^th ^percentileof MIC in a bacteria population; NCCLS: National Committee for Clinical Laboratory Standards; PA: *Pseudomonas aeruginosa*; PD: pharmacodynamics; PTA: probability of target attainment; qid: dose every 6 hours; SA: *Staphylococcus aureus*; T > MIC: time that antibiotic concentration is above bacteria MIC; tid: dose every 8 hours.

The time of infusion of meropenem has also been shown to influence its T > MIC. In a cross-over study of nine Thai VAP patients [20], after 48 hours of therapy, 1 g of meropenem tid in 30-minute infusions provided an adequate T > MIC in 74.7% of the patients, for a MIC of 1 mg/L. However, with a MIC of 16 mg/L, only the meropenem regimen of 2 g tid given in an extended infusion (two hours) led to a T > MIC > 40% [20].

Meropenem tissue PK have been evaluated by microdialysis in several studies (Table 3). The tissue-to-plasma meropenem mean ratio on the first day of antibiotic therapy was found to be 0.74 in the peritoneum [25] and 0.44 in subcutaneous fat [24]. The meropenem CFR was calculated for the 10 patients for whom serum levels were measured in this study according to the Mystic microbiological database [26]. The CFRs were 100% for Enterobacteriaceae and 40.6% for *Pseudomonas aeruginosa *after bolus dosing, whilst with continuous infusion they were 100% for both bacteria, despite the use of a small daily dose (2 g/day) [27].

**Table 3:** Tissue penetration of β-lactams^a^

Antibiotics	Samples	Patient demographics	Concentration ratios^b^	References
Muscle and subcutaneous tissue				
Meropenem	Microdialysis in subcutaneous tissue	*N *= 10 severe sepsis, 5 continuous infusion	Bolus 0.44 Continuous infusion 0.57 (day 2)	Roberts *et al*., 2009 [24]
Imipenem	Microdialysis in muscle and subcutaneous tissue	*N *= 11 (6 patients) Severe sepsis	Patients	Tegeder *et al*., 2002 [32]
			• Muscle 0.1	
			• Subcutaneous 0.14	
			Volunteers	
			• Muscle 0.5	
			• Subcutaneous 0.43	
Imipenem	Microdialysis in muscle	*N *= 12 (6 patients) Severe sepsis	Patients 1 Volunteers 0.97	Dahyot *et al*., 2008 [33]
Piperacillin	Microdialysis in muscle and subcutaneous tissue	*N *= 12 (6 patients) Septic shock	Patients	Joukhadar *et al*., 2001 [44]
			• Muscle 0.19	
			• Subcutaneous 0.1	
			Volunteers	
			• Muscle 0.55	
			• Subcutaneous 0.31	
Piperacillin	Microdialysis in subcutaneous tissue	*N *= 13 Severe sepsis	Bolus 0.21 Continuous infusion 0.2	Roberts *et al*., 2009 [45]
Cefpirome	Microdialysis in muscle	*N *= 18 (12 patients) Severe sepsis or septic shock	Patients 0.63 Volunteers 0.83	Joukhadar *et al*., 2002 [52]
Cefpirome	Microdialysis in subcutaneous tissue	*N *= 18 (11 patients) Severe sepsis	Patients 0.43 Volunteers 0.79	Sauermann *et al*., 2005 [51]
Burned skin				
Cefepime	Biopsy of burned area	*N *= 6 Burn patients	Day 3 1.52 (point concentration 3 to 5 hours after dose)	Sampol *et al*., 2000 [61]
Peritoneum				
Meropenem	Microdialysis in peritoneum	*N *= 6 Surgical peritonitis	0.74	Karjagin *et al*., 2008 [25]
Ceftazidime	Peritoneal drainage	*N *= 18 Surgical peritonitis	Day 2 • Continuous infusion 0.56	Buijk *et al*., 2002 [74]
			• Bolus 0.35	
Imipenem	ELF (bronchoscopy)	*N *= 8 Pneumonia	0.20 (point concentration ratio 2 hours after dose)	Muller-Serieys *et al*., 1987 [35]
Imipenem	Bronchial secretions (tracheal aspirate)	*N *= 10 Trauma patients with VAP	NR	McKindley *et al*., 1996 [34]
Piperacillin	ELF (bronchoscopy)	*N *= 10 VAP	0.57 (point concentration ratio 5 hours after dose)	Boselli *et al*., 2004 [41]
Piperacillin	ELF (bronchoscopy)	*N *= 40 VAP	0.44 (point concentration ratio 4 hours after dose)	Boselli *et al*., 2008 [43]
Piperacillin	Bronchial secretions (tracheal aspirate)	*N *= 8 VAP	0.36	Jehl *et al*., 1994 [42]
Cefepime	ELF (bronchoscopy)	*N *= 20 VAP	1.04 (point concentration ratio)	Boselli *et al*., 2003 [63]
Cefepime or ceftazidime	Bronchial secretions (tracheal aspirate)	*N *= 5 cefepime VAP	Cefepime < 0.02	Klekner *et al*., 2006 [62]
		*N *= 4 ceftazidime VAP	Ceftazidime < 0.05	
Ceftazidime	Bronchial secretions (tracheal aspirate)	*N *= 5 Pneumonia	0.12	Langer *et al*., 1991 [76]
Ceftazidime	Bronchial secretions (tracheal aspirate)	*N *= 12 Nosocomial pneumonia	0.76	Bressolle *et al*., 1992 [77]
Ceftazidime	ELF (bronchoscopy)	*N *= 15 VAP	0.21 (point concentration ratio at steady state)	Boselli *et al*., 2004 [69]

^a^ELF: epithelial lining fluid; NR: not reported. ^b^Mean area under the concentration time curve (AUC) tissue-to-plasma ratio unless otherwise stated.

#### Imipenem

In ICU patients, increased *V*_d _and Cl of imipenem have also been reported (Table 1). Therefore, its T_1/2 _and T > MIC may be difficult to predict, depending on the relative changes of these two parameters. This difficulty was shown by Belzberg *et al*. [28] in a cohort of ICU surgical and trauma patients with presumed Gram-negative sepsis. In this relatively young population (mean age 45.2 ± 17 years and mean body weight 79.7 ± 17.7 kg), 44% of patients presented trough levels lower than the intended 4 mg/L at steady state. A mean Cr Cl of 103.8 mL/minute was found, but with large variability: two patients had renal failure and nineteen patients had a Cr Cl > 120 mL/minute. Nevertheless, no correlation was found between PK parameters and body weight, severity of disease, blood pressure or renal function [28].

Another study compared meropenem and imipenem first-dose PK in patients with normal renal function (serum Cr < 1.5 mg/dL). Again, both *V*_d _and Cl were significantly elevated, although more so in the meropenem group [29]. However, their T > MIC for sensitive isolated pathogens were similar. Again, there was a relationship between Cr Cl and T_1/2_: Patients with a Cr Cl < 50 mL/minute had a significantly longer T_1/2 _for both antibiotics.

The PD efficacy of imipenem is also influenced by the dose and the time of infusion [30]. Using PK data from a cross-over steady-state study of VAP patients, Jaruratanasirikul and Sudsai [30] showed by modelling of imipenem PD that, for a MIC of 4 mg/L, a 500-mg dose delivered every 6 hours (qid) for 30 minutes achieved a T > MIC of 64.7% and increased to 76.5% with a 2-hour infusion. However, this study excluded shock and renal failure patients (Cr Cl < 60 mL/minute). With PD modelling of PK data derived from another 20 VAP patients [31], continuous infusion led to improved PTA despite the use of lower dosages (Table 2). In this latter study, all patients had *f *imipenem T > MIC of 100%, but three patients died.

Tissue microdialysis had been used to assess imipenem PK, but with very dissimilar results (Table 3): namely, the tissue-to-plasma ratio. This has been found to be markedly depressed in a cohort of severe critically ill patients compared to healthy volunteers (subcutaneous tissue-to-plasma 0.14 vs. 0.43 and muscle tissue-to-plasma 0.11 vs. 0.5, respectively) [32]. However, Dahyot *et al*. [33] disputed these results and found *f *imipenem in plasma and muscle to be virtually superimposed at any time, both in patients and in healthy volunteers. Some differences exist between these two studies. In the Tegeder *et al*. study [32], the patients had lower Cr Cl (medians 32.8 mL/minute vs. 156 mL/minute) and samples were collected at steady state and not after the first dose. Moreover, Dahyot *et al*. [33] accounted only for the *f *imipenem in plasma and found higher imipenem *V*_d _and Cl. Different methods of calculating *in vivo *microdialysis recovery rates may also explain some of the diverse observed results. Nevertheless, low imipenem penetration ratios, as low as 0.06 [34,35], in bronchial secretions were reported in pneumonia patients (Table 3).

### Penicillins

#### Piperacillin

Similarly to other β-lactams, piperacillin *V*_d _and Cl have generally been found to be increased in ICU patients (Table 1). However, most studies have excluded renal failure patients.

Piperacillin Cl and trough concentrations were strongly related to Cr Cl [36-38]. Taccone *et al*. [23] showed that only 15% of patients with high Cr Cl (> 50 mL/minute) maintained piperacillin concentrations > 50% of T > 4 × MIC after the first antibiotic dose, as opposed to 71% of patients with lower Cr Cl (*P *= 0.03). In contrast, in 10 young burn patients (mean total burned area 40.8 ± 3.1%) with a mean Cr Cl of 119.8 mL/minute and *Pseudomonas aeruginosa *infection, the authors found a 20% increase in T_1/2 _after the first dose of antibiotic compared to the third day of therapy, which was related to a larger *V*_d _(mean of 19.6 L vs. 16.4 L) [38]. Overall, the piperacillin AUC was similar in the two measurements (mean of 640 mg × hour/L vs. 622 mg × hour/L).

Piperacillin is stable for at least 24 hours at room temperature, making it a suitable choice for continuous infusion. With this strategy, higher steady-state concentrations are expected, theoretically providing a higher T > MIC even with the use of a lower daily dose [39]. A study by Rafati *et al*. [40] also supports this strategy. These authors showed that, for a MIC = 16 mg/L, the T > MIC was higher with continuous infusions (8 g/day) than with bolus dosing (3 g tid) (100% vs. 62%, respectively). However, the mortality rate was similar.

In VAP patients, piperacillin showed good penetration in bronchial secretions [41-43]. Nevertheless, its epithelial lining fluid (ELF) steady-state concentration was lower than the MIC for *Pseudomonas aeruginosa *after a 4.5-g tid dose [41]. With continuous infusion, an increase in pulmonary concentration was found, at least in the subset of patients with moderate renal failure (measured Cr Cl < 50 mL/minute), about three times higher than in the patients with normal renal function [43]. However, no relationship was found between ELF piperacillin concentration and clinical success. Similar concentrations were found in the eight patients who died or had persistent infections and in those who experienced therapeutic success [43].

Subcutaneous tissue-to-plasma ratio and PK have been assessed in microdialysis studies. In six septic shock patients (mean norepinephrine dose 0.8 μg/kg/minute) [44], the subcutaneous tissue-to-plasma AUC ratio was only 0.1, one-third of that measured in healthy volunteers. Peak tissue concentration was also delayed in patients (122 minutes in patients compared with 27 minutes in healthy volunteers), and T_1/2 _in tissues was nearly nine times longer. In 13 younger patients with less severe sepsis [45], the AUC tissue-to-plasma ratio was roughly 0.2. In accordance with their serum PK (as well as PK of another five patients) [46], piperacillin/tazobactam CFR was calculated to be 92.3% with continuous infusion (13.5 g/day) and 53.4% with bolus dosing (4.5 g qid, or 18 g/day). Again, no correlation was found between tissue concentration and outcomes. Despite the low tissue concentration levels, all patients in both groups survived [45].

### Cephalosporins

#### Cefpirome

Cefpirome PK studies have produced heterogeneous results. A 2-g dose was adequate in young trauma patients (Cr Cl ≥ 50 mL/minute) and in similar healthy volunteers. After the first dose, the mean T > MIC were 75% and 80%, respectively (with a MIC of 4 mg/L, *P *= 0.76) [47]. However, in 12 similar patients, a lower T > MIC (60%) was found, which was probably related to higher cefpirome Cl [48]. After four days of therapy, the cefpirome mean PK parameters remained similar (T > MIC 67% and AUC 242 mg × hour/L vs. 306 mg × hour/L at steady state). Further analyses [49] showed a strong correlation between Cr Cl and either cefpirome or cefepime Cl (*r*^2 ^= 0.81). Patients with the lower range of T > MIC had a higher Cr Cl, usually above 144 mL/minute [49]. According to these measured PK data, the authors performed a simulation to demonstrate improved CFR of cefpirome given as a continuous infusion to treat *Pseudomonas aeruginosa *infection, from 56.1% to 84.4% (Table 2) [50].

Cefpirome tissue PK were evaluated on the basis of microdialysis. Sauermann *et al*. [51] found a low subcutaneous tissue concentration in patients with severe sepsis, almost half of healthy volunteers, despite a longer plasma T_1/2 _(183 minutes vs. 95 minutes; *P *< 0.05). Similar results were reported by Joukhadar *et al*. [52], who found muscle-to-plasma ratios of 0.63 in patients and 0.83 in healthy volunteers (Table 3).

#### Cefepime

Roughly a twofold variation of cefepime *V*_d _has been reported in PK studies (Table 1) of severe sepsis and septic shock patients [23], elderly septic patients [53], young burn patients [54] and nosocomial pneumonia patients [55]. Cefepime Cl has also been found to be closely correlated with Cr Cl in this last listed cohort (*r*^2 ^= 0.77) [55], in another cohort of septic patients (*r*^2 ^= 0.74) [56] and in burn patients (*r*^2 ^= 0.58) [57]. Therefore, patients with renal dysfunction may experience toxicity.

In 21 septic patients receiving cefepime at a dose of 2 g bid, more than twofold peak variations and roughly 40-fold trough variations were observed. Again, the cefepime Cl correlated with Cr Cl (*r*^2 ^= 0.77). Two patients with low Cr Cl (19 and 12 mL/minute) had trough levels > 20 mg/L despite dosage adjustment. They both had neurologic symptoms (namely, confusion and muscle jerks) that were not identified as toxicity but resolved promptly after drug arrest [55].

A cefepime bolus of 2 g bid was found to be insufficient to reach a high PD target after the first dose (Table 2), both in 80% of young burn patients (burn area 21.8%) with high mean Cr Cl (119.2 mL/minute) [54] and in the Taccone *et al*. study [23], in which only 16% of patients achieved the intended target.

Two other studies have evaluated cefepime PK, one of which addressed the first day of therapy for 55 nosocomial pneumonia or bacteraemia patients (67% trauma) [58] and the other of which described the status of 32 VAP patients on the second day of cefepime treatment [59]. Both studies unveiled a relationship between *V*_d _and total body weight as well as between excretion, either elimination rate constant [59] or Cl [58], and Cr Cl. However, significant interpatient variability was again observed, with regard to both cefepime Cl (58%) and central compartment *V*_d _(67%) [58].

A PD model was developed with this VAP population PK data: despite a 2-g tid dose, PTA > 90% was achieved only with a MIC ≤ 8 mg/L [59]. In another cefepime PD model, the CFR of a 2-g bid dose, used to treat both *Escherichia coli *and *Klebsiella pneumoniae*, was 78.9%. However, for *Pseudomonas aeruginosa*, CFR was only 53.6% (Table 2) and increased with either 2 g tid or continuous infusion (4 g/day or 6 g/day) to 84.9%, 91.7% and 94.8% respectively. Nevertheless, the CFR for *Acinetobacter baumanii *[60], even with a continuous infusion of 6 g/day, was only 75%, reemphasizing the importance of appropriate dosing and the potential benefit of continuous infusion against difficult-to-treat bacteria.

Also, the cefepime tissue concentration was assessed in biopsy samples collected from the skin of burn patients three to five hours after a bolus dose on day 3 of antibiotic therapy. A mean biopsy-to-plasma cefepime ratio of 1.5 (range 0.4 to 5.1) was found [61]. Klekner *et al*. [62] were unable to detect cefepime in bronchial secretions from any of the five studied patients six hours after an 80 mg/kg dose. However, using continuous infusion (4 g/day) to treat VAP patients, Boselli *et al*. [63] found, at steady state, higher and similar plasma and ELF concentrations (mean of 13.5 mg/L and 14.1 mg/L, respectively). Although different sampling methods may have influenced these differences, continuous infusion seems to prolong T > MIC in the lungs. Nevertheless, no correlation with therapeutic outcomes was reported.

#### Ceftazidime

Several studies have shown ceftazidime PK heterogeneity in ICU septic patients with *Pseudomonas *infections (mostly nosocomial pneumonia) [64], severe sepsis [65,66] and burns [54]. Similarly to other β-lactams, the authors noted a large variation of both *V*_d _and Cl (Table 1) and consequently significant interpatient variability in T_1/2 _and trough concentrations. Also, a correlation between Cl and Cr Cl was usually reported [65,66].

Continuous infusion of ceftazidime was compared with bolus dosing in five different studies [67-71]. In all, there was an increase in T > MIC with continuous infusion despite lower daily doses. However, only in severe melioidosis was this strategy associated with lower mortality (3 of 10 patients vs. 9 of 11 patients) [71]. Those patients had low Cr Cl (26.6 mL/minute) and received ceftazidime dosages adjusted to their body weight (4 mg/kg/hour or 40 mg/kg tid, for a mean body weight of 49.4 kg). Ceftazidime steady-state concentration was measured in another cohort of 92 patients receiving continuous infusions [72]. Therapeutic drug monitoring was performed on the second day of therapy. The mean serum concentration was 46.9 mg/L, but again with a very wide range of serum concentrations (7.4 to 162.3 mg/L). Therefore, dosage modification was common because of low serum levels (36.9%) and high serum levels (27.2%), with the latter being associated with lower Cr Cl (mean of 51 mL/minute compared with 103 mL/minute for patients with low serum levels). Similar results were shown in another large ceftazidime PK study assessing a mixed septic population with a higher mean Cr Cl (123 mL/minute) [73]. The lower T > MIC was found in patients with the higher Cr Cl, especially after bolus dosing (Table 2).

Continuous infusion of ceftazidime (4.5 g/day) was also associated with a higher peritoneal AUC at day 2 compared to bolus dosing (1.5 g tid) in surgical patients with peritonitis (522 mg × hour/L vs. 316 mg × hour/L; *P *= 0.01) [74], despite similar serum AUC (and Cr Cl > 30 mL/minute). Therefore, although serum T > 4 × MIC was > 90% in all patients, peritoneal T > 4 × MIC was still > 90% with continuous infusion but only 44% with bolus dosing. Nevertheless, no difference in mortality was noted (25% vs. 33%; *P *= 1.0). A PD model of ceftazidime in ICU patients also showed higher PTA with continuous infusion (100% for MIC ≤ 8 mg/L) than with bolus dosing [75].

Ceftazidime concentration in bronchial secretions was measured in four studies of VAP patients. Very low concentrations, < 0.5 mg/L and < 0.3 mg/L, were found in two of them [62,76]. Bressole *et al*. [77] found a higher ratio between bronchial secretions and plasma concentration (0.76) in patients infected after abdominal surgery. A longer T_1/2 _(6.1 hours) and a lower Cl (4.2 L/hour) may explain some of these differences. With continuous infusion, a ratio of 0.21 between ELF and serum was observed [69].

## Discussion

In our systematic review, we have aggregated information from 57 prospective studies related to the PK of β-lactam antibiotics, which are among the most often agents used to treat sepsis in ICU patients [78]. Overall, an increased *V*_d _of all the studied antibiotics was reported (Figure 3), which was related to total body weight [58,73], but with significant variability. Drug Cl was also increased and usually related to Cr Cl. Those changes were largely unpredictable, with important interpatient variability. However, the higher Cl values were noted in studies that excluded patients with renal dysfunction, a common strategy, which may limit the interpretation of the data reported.

Therapeutic drug monitoring was rarely performed. In addition, data on the daily variation of PK parameters in ICU patients, as well as the ideal frequency of this monitoring, are currently limited. Nevertheless, two of the reviewed studies [55,72] showed that inadequate dosing may be common in this population and may jeopardize β-lactam antibiotics efficacy or even lead to toxicity [79]. Roberts *et al*. [80] measured piperacillin/tazobactam concentrations and found that 50.4% of patients first measurement were low. The clinical efficacy of using drug levels to achieve adequate concentrations had never been properly evaluated. In a recent study, PD modelling was used to empirically treat 94 VAP in critically ill patients at high risk of infection with antibiotic-resistant *Pseudomonas aeruginosa *[81]. A three-hour infusion regimen of either cefepime or meropenem at a high dosage (2 g tid) was initiated, followed by both antibiotic and dose de-escalation whenever bacteria with a low MIC were identified. The infection-related mortality decreased from 21.6% to 8.5% (*P *= 0.029).

The PD targets of β-lactam antibiotics may be different in patients with severe bacterial infections. McKinnon et al. [82] evaluated ceftazidime and cefepime PD by using PK data from previous clinical trials [83]. Maintaining a T > MIC as high as 100% was associated with a significantly greater clinical cure and bacteriologic eradication than a shorter time (cefatzidime: 82% vs. 33%, P = 0.002; cefepime: 97% vs. 44%, P = 0.001). Also, in a febrile neutropenia population of 60 patients treated with meropenem, a calculated T > MIC of 83% was found in responders, whilst those with a poor clinical response had a T > MIC of only 60% [84]. It has also been suggested that, at least in vivo, maximum killing of bacteria is achieved at higher concentrations, four to five times MIC [85], accounting for antibiotic penetration in infected tissues. As such, concentrations of β-lactam antibiotics may need to be maintained well above the MIC for extended periods, especially in patients with life-threatening infections. Accordingly, different PD targets have been proposed in the different studies addressing ICU patients, which sometimes make their comparison difficult.

An improved PD profile of β-lactams may be obtained by promoting a longer exposure with more frequent dosing, extended infusions or continuous infusions [86,87]. Several of the studies that we reviewed reported PD benefits of continuous infusions (even using small daily doses) (Table 2). Also, PD modelling tends to support this strategy. Nevertheless, almost none of the studies addressed reported a decrease in mortality. In addition, a recently published meta-analysis of 14 prospective studies did not show a significant benefit of using this strategy (odds ratio 1.00, 95% confidence interval 0.48 to 2.06; *P *= 1.00) [88].

An increasing number of studies have addressed β-lactam antibiotic tissue concentration. Despite the theoretical advantage of analysing the drug concentration at the site of infection, there are no data to support a relationship between these concentrations and outcomes. Furthermore, there are still controversial issues involved in interpreting these data, namely, microdialysis [32,33]. Therefore, we think that, at present, no recommendation can be made regarding antibiotic tissue PK.

## Conclusions

The PK of β-lactam antibiotics are significantly changed in septic ICU patients. Dosage and schedule regimens based on data from healthy volunteers may be misleading. Therapeutic drug monitoring and PD modelling according to measured PK previously showed promising results. Continuous infusion, although theoretically useful, has not been shown to lead to improved outcomes. The clinical significance of tissue PK monitoring remains to be determined.

## Key messages

• Among ICU patients, the PK of β-lactam antibiotics are markedly unpredictable.

• A large volume of distribution is commonly observed in ICU patients and contributes to a lower antibiotic concentration, but also to a greater exposure time.

• An increased glomerular filtration rate is usually associated with a short half-life of β-lactam antibiotics, whilst renal failure is associated with a greater exposure and increased risk of accumulation.

• Continuous infusion of β-lactam antibiotics commonly increases the time that the antibiotic concentration exceeds its MIC and may therefore increase efficacy.

• Therapeutic drug monitoring of β-lactam antibiotic concentration may help to improve its efficacy and prevent toxicity, but currently is unavailable in most clinical settings.

## Abbreviations

AUC: area under the concentration time curve; bid: dose every 12 hours; CFR: cumulative fraction of response; Cl: drug clearance; Cr Cl: creatinine clearance; ELF: epithelial lining fluid; *f*: free drug fraction; *K*_el_: elimination rate constant; MIC: minimum inhibitory concentration; MIC_90_: 90^th ^percentile of MIC in a bacteria population; PD: pharmacodynamics; PK: pharmacokinetics; PTA: probability of target attainment; qid: dose every six hours; T_1/2_: half-life; tid: dose every eight hours; T > MIC: antibiotic concentration time over bacteria MIC; VAP: ventilator-associated pneumonia; *V*_d_: volume of distribution.

## Competing interests

JGP has received honoraria from and served as an advisor for Pfizer, AstraZeneca, Gilead Sciences Inc., Abbott Laboratories, Wyeth Lederle, Janssen-Cilag and Merck Sharp & Dohme Corp. JGP also has received an unrestricted research grant from AstraZeneca. PP has received honoraria from and served as an advisor for AstraZeneca, Ely Lilly and Co., Gilead Sciences Inc., Janssen-Cilag, Merck Sharp & Dohme Corp., Novartis and Pfizer Inc.

## Authors' contributions

Both JGP and PP searched the literature, analysed the data and wrote the manuscript. Both authors read and approved the final manuscript for publication.
